# Ribosomal RNA modification enzymes stimulate large ribosome subunit assembly in *E. coli*

**DOI:** 10.1093/nar/gkae222

**Published:** 2024-03-30

**Authors:** Rya Ero, Margus Leppik, Kaspar Reier, Aivar Liiv, Jaanus Remme

**Affiliations:** IMCB University of Tartu, Riia 23, 51010 Tartu, Estonia; IMCB University of Tartu, Riia 23, 51010 Tartu, Estonia; IMCB University of Tartu, Riia 23, 51010 Tartu, Estonia; IMCB University of Tartu, Riia 23, 51010 Tartu, Estonia; IMCB University of Tartu, Riia 23, 51010 Tartu, Estonia

## Abstract

Ribosomal RNA modifications are introduced by specific enzymes during ribosome assembly in bacteria. Deletion of individual modification enzymes has a minor effect on bacterial growth, ribosome biogenesis, and translation, which has complicated the definition of the function of the enzymes and their products. We have constructed an *Escherichia coli* strain lacking 10 genes encoding enzymes that modify 23S rRNA around the peptidyl-transferase center. This strain exhibits severely compromised growth and ribosome assembly, especially at lower temperatures. Re-introduction of the individual modification enzymes allows for the definition of their functions. The results demonstrate that in addition to previously known RlmE, also RlmB, RlmKL, RlmN and RluC facilitate large ribosome subunit assembly. RlmB and RlmKL have functions in ribosome assembly independent of their modification activities. While the assembly stage specificity of rRNA modification enzymes is well established, this study demonstrates that there is a mutual interdependence between the rRNA modification process and large ribosome subunit assembly.

## Graphical Abstract

**Graphical Abstract:**
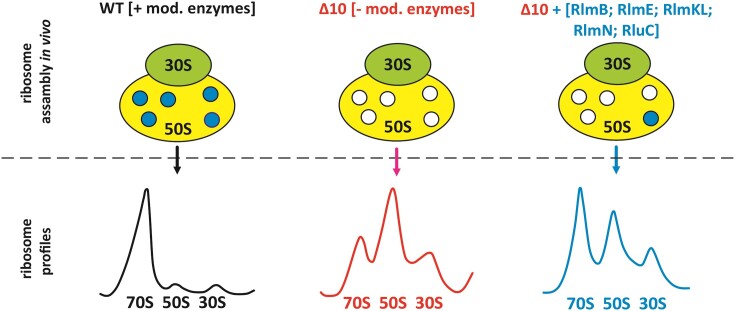

## Introduction

Ribosomes from all three domains of life exhibit conservation of core structural and functional features (1). The 70S ribosomes of bacteria are made up of a large (LSU, 50S) and a small (SSU, 30S) subunit composed of roughly two-thirds ribosomal RNA (rRNA) and one-third proteins (r-proteins). Post-transcriptional modification of rRNA is an integral and ubiquitous part of ribosome synthesis. In all organisms, specific sets of rRNA nucleosides are covalently modified during ribosome biogenesis. Pseudouridines (Ψ) and various methyls represent the two major types of rRNA modifications. There is a correlation between the overall complexity of an organism and the number of ribosome modifications, mostly pseuoduridines and 2′*O* methyls (2–4).

**Figure 1.**
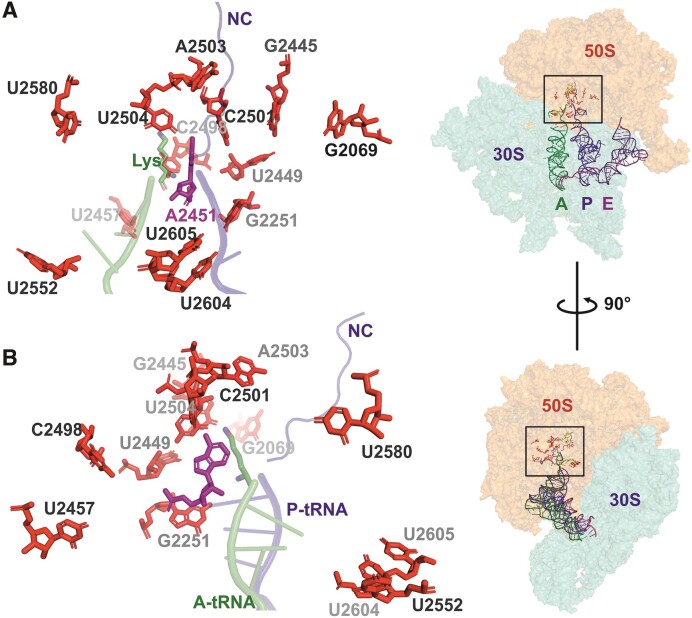
Position of 23S rRNA domain V residues that are post-transcriptionally modified in E. coli. Panels A and B are close-up views of the PTC region (view rotated 90° around the y-axis as shown in right). Insert: A-, P- and E-site tRNAs are depicted as cartoons, while subunit rRNAs are depicted as transparent surfaces. (**A** and **B**) The 3' CCA region of A-site tRNA charged with lysine (Lys) and P-site tRNA carrying a nascent chain (NC) are shown in green and blue, respectively. 23S rRNA residue A2451 (magenta sticks) participates in the catalysis of the peptide bond. Post-transcriptionally modified residues in *E. coli* 23S rRNA (Table 1) are shown in red sticks. The figure is based on the structure of an ErmBL-stalled ribosome (PDB ID: 5JTE) (76).

Most modified nucleosides (MN) are located near the functionally important regions of the ribosome (5). The peptidyl-transferase center (PTC), located in the LSU and consisting predominantly of RNA (23S rRNA domain V), catalyzes the key reaction of protein synthesis. Specifically, the formation of the peptide bond between the amino acid attached to the tRNA in the aminoacyl-site (A-site) and the nascent peptide chain attached to the tRNA in the peptidyl-site (P-site). The main components of the bacterial PTC are the 23S rRNA elements: A-loop (in helix 92), P-loop (in helix 80), and the nucleotides around the entrance of the nascent peptide exit tunnel including residue A2451 (*Escherichia coli* numbering hereafter). The 3′ CCA ends of the ribosome A-site-bound aminoacyl-tRNA and P-site-bound peptidyl-tRNA interact with the PTC A- and P-loops, respectively (6–8). PTC is linked to the nascent peptide exit tunnel, which provides the nascent chain a passage through the LSU. Out of the 36 naturally occurring (also known as ‘housekeeping’) MN in *E. coli*, 13 are located around the PTC (in 23S rRNA domain V within 25 Å of the catalytically essential A2451 residue). These include five pseudouridines (Ψ2457, Ψ2504, Ψ2580, Ψ2604 and Ψ2605), three 2'O ribose (Gm2251, Cm2498 and Um2552), and three base (m^7^G2069, m^2^G2445 and m^2^A2503) methylations, as well as one dihydrouridine (D2449) and one 5-hydroxycytidine (ho^5^C2501) (9) (Table 1 and Figure 1). In bacteria, each rRNA MN is made by a specific modification enzyme(s) (ME); however, some MEs are responsible for synthesizing the same type of MN at more than one position in rRNA (Table 1). For instance, RluC synthesizes Ψ2504 and Ψ2580 in the PTC region, as well as Ψ955 located further away (10). In the case of the *E. coli* RlmKL protein, m^7^G2069 is first introduced to 23S rRNA by its RlmK domain, followed by m^2^G2445 synthesis by its RlmL domain. Separate RlmK and RlmL methyltransferases are found in other bacteria (11). *E. coli* RlmN is a dual-specificity enzyme that, in addition to synthesizing m^2^A2503 in 23S rRNA, is also responsible for m^2^A37 in 6 tRNA species (12). Likewise, RluF catalyzes the synthesis of Ψ2604 in 23S rRNA as well as Ψ35 in the anticodon of tyrosine tRNA (13). Most of the PTC region MNs are synthesized during the early or intermediate stages of ribosome assembly, whereas RlmE appears to synthesize Um2552 late in ribosome biogenesis (14,15). The gene encoding RldA is not known. However, the growth and antibiotic sensitivity of cells expressing only the mutant D2449C 23S rRNA were indistinguishable from those of the wild-type strain (*WT*), suggesting that the D2449 modification is dispensable (16).

**Table 1.** Modified nucleosides in 23S rRNA domain V of *E. coli* ribosome

23S rRNA position	Modification	Distance to A2451 2′O (Å)	Enzyme
2069	m^7^G	23.9	RlmKL (RlmK domain)
2251* (P-loop)	Gm	13.5	RlmB
2445	m^2^G	12.3	RlmKL (RlmL domain)
2449	D	16.5	RldA (gene unknown)
2457*	Ψ	19.5	RluE
2498	Cm	20.2	RlmM
2501*	ho^5^C	11.6	RlhA
2503	m^2^A	18.6	RlmN
2504	Ψ	12.9	RluC
2552* (A-loop)	Um	23.3	RlmE
2580	Ψ	21.7	RluC
2604	Ψ	20.1	RluF
2605	Ψ	21.6	RluB

*Evolutionary conserved (from bacteria to humans) modification site.

Curiously, regardless of their ubiquitous presence, clustering around the functional sites of the ribosome, and metabolic burden of their synthesis, not a single MN of rRNA has been found to be essential for bacterial survival. Indeed, the vast majority of *E. coli* rRNA MEs can be knocked out individually with minimal, if any, decrease in growth rate (10,17–20). Only the RlmE knockout strain (*ΔrlmE*) exhibits a notable (2–4-fold) decrease in growth rate compared to *WT E. coli* cells (21–23). RlmE is responsible for the synthesis of Um2552, situated adjacent to G2553, which is an essential base that anchors the 3' CCA terminus of the A-site tRNA in the PTC (24,25) (Table 1 and Figure 1). The severe assembly phenotype of *ΔrlmE* can be restored by the overexpression of two different families of small GTPases (Obg and EngA), suggesting that RlmE has a function in ribosome LSU assembly independent of its methylase activity. Moreover, it refers to a functional redundancy between RNA MEs and certain small GTPases (26). A single deletion strain of other 23S rRNA domain V-specific modification enzymes does not exhibit ribosome assembly defects (20). Aside from Um2552, the lack of several MNs simultaneously might cumulatively have an effect on the ribosome and thereby on bacterial fitness. However, the *E. coli* multiple knockout (*ΔrluC*, *ΔrlmKL*, *ΔrlmN*, *ΔrlmM* and *rluE*) strain, whose ribosomes lack a total of 7 PTC region MNs, is viable and exhibits only a modest increase in doubling time at 37°C (27). Furthermore, the simultaneous deletion of seven pseudouridine synthases, preventing the synthesis of all 11 Ψ in rRNA, results in only a small reduction in *E. coli* growth rate under a variety of growth conditions (28). Interestingly, unspecific isomerization of uridines affects large ribosome subunit assembly in a position-dependent way (29) demonstrating the potential of Ψs to influence ribosome assembly. Loss of Ψs in rRNA is surprisingly well tolerated in bacteria but severely impairs ribosome assembly and function in eukaryotes (30). In eukaryotes, it was found that deleting individual pseudouridines has no or only slight effects on growth or ribosome activity, whereas multiple deletions have cumulative negative effects that can be synergistic (31,32).

Modifications are known to change the conformation and chemical properties of the corresponding RNA regions. For instance, rRNA MNs can increase rRNA base stacking interactions and stabilize RNA helices, as well as alter hydrogen bonding donor and acceptor patterns (33–37). However, the exact relevance of the vast majority of the MNs remains elusive. Based predominantly on the fact that the corresponding ME deletion strains lack notable phenotypes, a ‘fine-tuning’ function that is difficult to pinpoint and quantify has been proposed for rRNA MN in bacteria (2,5,20).

Ribosome assembly is fast and efficient, taking only 2–3 min at 37°C (38). In recent publications, 50S early assembly intermediates and the 23S rRNA folding pathway during ribosome assembly *in vivo* (39) and *in vitro* (40,41) were described. Structural transitions are energized by ribosomal proteins and non-ribosomal assembly factors (39). RNA helicases, ribosome binding proteins, protein chaperones, and small GTPases stimulate ribosome assembly, in particular at lower temperatures (for review (42)). As mentioned above, RlmE is among the factors needed for fast and efficient 50S assembly. The importance of other MEs and MNs is not known. This paper partially fills this information gap.

In this study, we aimed to design a genetic background to facilitate the functional studies of ribosomal RNA modification. We constructed an *E. coli* strain lacking 10 rRNA MEs (*ΔrlmB*, *ΔrlmE, ΔrlmKL*, *ΔrlmM*, *ΔrlmN*, *ΔrluB*, *ΔrluC*, *ΔrluE*, *ΔrluF* and *ΔrlhA*), resulting in the absence of modifications around the PTC (Table 1 and Figure 1). The *Δ10* strain struggles to grow due to defective ribosome assembly, which makes it a tool for assessing the role of individual PTC MNs and respective MEs in ribosome biogenesis by ectopic expression of corresponding MEs. We show that several rRNA MNs, foremost the ones synthesized by RluC (Ψ955, Ψ2504 and Ψ2580), RlmKL (m^7^G2069 and m^2^G2445), RlmN (m^2^A2503), and RlmB (Gm2251), in addition to RlmE (Um2552), significantly contribute to bacterial fitness by facilitating ribosome assembly. RlmB and RlmL stimulate ribosome LSU assembly independent of their modification activity.

## Materials and methods

### Bacterial strains and plasmids

Deletions were introduced sequentially into the *E. coli* MG1655 (F- lambda- *ilvG*- *rfb*-50 *rph*-1) K-12 strain by P1-mediated transduction to generate the *Δ9* and *Δ10* multiple rRNA ME knockout strains essentially as described in (27). The order of gene knockouts was performed as follows: *ΔrluC*, *ΔrlmKL*, *ΔrlmN*, *ΔrlmM*, *ΔrluE*, *ΔrluB*, *ΔrluF*, *ΔrlmB*, *ΔrlhA* and *ΔrlmE*. The difference between the *Δ9* and *Δ10* strains lies in the lack of the *rlmE* gene in the latter. The precursor single knockout strains were from the KEIO collection (17). *ΔrluE* was constructed according to the method (43). In between each subsequent deletion step, the kanamycin resistance cassette was removed as described in (44).

Modification enzyme expression plasmids were constructed for this study based on the vector pHBT (a derivate of pHSG576) (45). pHBT is a low copy number plasmid (3–5 copies per cell) containing the pCS101 origin, chloramphenicol (Cam) selection marker, and *tac* promoter. pHBT-based vectors exhibit leaky expression in the absence of isopropyl β-d-1-thiogalactopyranoside (IPTG), resulting in low levels of the corresponding MEs. ME genes were amplified from *E. coli* genomic DNA and inserted into pHBT vector between BamHI and KpnI restriction sites. Mutations were introduced into pHBT-*rluC* (resulting in RluC-D144A), pHBT-*rlmKL* (RlmKL-N309A), pHBT-*rlmL* (RlmL-N309A), pHBT-*rlmN* (RlmN-C355A) and pHBT-*rlmB* (RlmB-E198A) plasmids with site-directed mutagenesis using Phusion DNA polymerase (Thermo Scientific) by inverse PCR. Combined polynucleotide kinase (PNK), DpnI, and T4 DNA ligase (all from Thermo Scientific) were used in one step following the PCR approach to generate the plasmids according to the manufacturer's instructions. Expression vectors were verified by sequencing.

### Bacterial growth

Modification enzyme expression vectors were transformed into chemo-competent Δ10 or Δ9 cells using heat shock and cultivated on LB (10 g tryptone, 5 g yeast extract, and 10 grams NaCl *per* 1 liter distilled water with pH adjusted to 7.0) plates supplemented with Cam (15 μg/ml) at 37°C overnight. An empty pHBT plasmid was used for control. For reference, *E. coli* MG1655 (no antibiotic resistance selection) and Δ10 or Δ9 strains (in both cases, 50 μg/ml Km for selection) were used. 2 ml of fresh 2× YT (16 g tryptone, 10 g yeast extract, and 5 g NaCl *per* 1 l distilled water with pH adjusted to 7.0) or LB media supplemented with relevant antibiotics was inoculated with a single colony and grown to mid-log phase. Dilutions (final OD_600_ 0.01) were made into 150 μl LB media in CellStar (Greiner bio-one) 96-well suspension culture plates with or without 1 mM IPTG. No antibiotics were added. Plates were covered with clear tape and incubated at 30°C or 37°C (with continuous shaking at 500 rpm). OD_580_ was determined using BMG Labtech POLARstar Omega every 7 min for up to 30 h. Doubling time (g) was calculated from the early log phase [*g* = ln 2/*r*, where *r* = ln(*N*/*N*_0_)/(*t* – *t*_0_)]. Average doubling times and standard deviations were calculated from at least three biological replicates with at least three technical replicates each.

### Sucrose density gradient centrifugation

100 ml of 2× YT media supplemented with relevant antibiotics and inoculated with overnight bacterial cultures were grown at 25°C, 30°C, or 37°C to an OD_600_ of ∼0.5 (early log phase) or ∼1.5 (mid log phase). Cells were collected by centrifugation (4000 rpm for 15 min at 4°C) and re-suspended in 1 ml LP lysis buffer (60 mM KCl, 60 mM NH_4_Cl, 50 mM Tris–HCl pH 8.0, 6 mM MgCl_2_, 16% sucrose and 6 mM 2-mercaptoethanol) supplemented with DNase I (10 U/ml). Cells were disrupted with glass beads using the Bertin Precellys24 Tissue Homogenizer (3 cycles of 60 s on/off 6000 rpm at 4°C). Lysate was clarified by centrifugation (13 000 rpm for 15 min at 4°C) and diluted with 1 ml LLP lysis dilution buffer (60 mM KCl, 60 mM NH_4_Cl, 10 mM Tris–HCl pH 8.0, 12 mM MgCl_2_ and 6 mM 2-mercaptoethanol). 50 (*A*_260_) units of lysate was layered onto 10% to 30% (w/w) sucrose gradient in OV buffer (60 mM KCl, 60 mM NH_4_Cl, 10 mM Tris–HCl pH 8.0 and 6 mM 2-mercaptoethanol) containing 10 mM MgCl_2_. Ultracentrifugation was carried out (ω^2^t = 3.0 × 10^11^ rad/s) at 4°C using a Beckman Coulter SW-28 rotor. Ribosome profile was determined by continuous monitoring of absorbance at 260 nm. Fractions corresponding to 70S ribosomes and free 50S subunits were collected. Ribosome profiles were quantified using the ImageJ program to determine the peak areas corresponding to the 70S, 50S and 30S fractions.

For Mg^2+^ dependency analysis, free 50S subunit fractions were concentrated, sucrose diluted, and Mg^2+^ adjusted to 0.5, 1.0 or 2.0 mM using Amicon Ultracel-100K centrifugal filters. Samples were loaded onto a 10–25% (w/w) sucrose gradient in OV buffer containing 0.5, 1.0 or 2.0 mM MgCl_2_, respectively. Ultracentrifugation was carried out (ω^2^*t* = 3.0 × 10^11^ rad/s) at 4°C, and ribosome profiles were recorded.

### Primer extension analysis

Processing of the 23S rRNA 5′ end was analyzed by primer extension. 70S ribosome and 50S subunit fractions from sucrose gradient centrifugation were pelleted by ultracentrifugation (ω^2^*t* = 1.2 × 10^12^ rad/s) using a Beckman Coulter Ti-50 rotor. Pellets were suspended in 200 μl OV buffer containing 10 mM MgCl_2_ and RNA was extracted using 800 μl of Buffer PM (Qiagen). Samples were vortexed for 20 min at room temperature. 20 μl of 50% SiO_2_ suspension was added, followed by shaking for an additional 10 min. The silica was pelleted by centrifugation at 13 000 rpm for 30 s. Pellet was washed twice with 70% ethanol, air-dried, and RNA was eluted by incubating the silica in water (Milli-Q) for 5 min at 55°C. Centrifugation was used to pellet the silica during the washing and elution steps. RNA concentration (*A*_260_) was measured and samples were stored at -20°C.

Reverse transcription from the 23S 5′ END primer (5′-TCG CCT CTG ACT GCC AGG GCA TCC-3′) with 5′ FAM modification (Microsynth) was used to map the 5′ end of 23S rRNA using AMV reverse transcriptase (Promega) essentially as described in (46). The resulting cDNA fragments were resolved in a 7% polyacrylamide-urea gel and fluorescence was visualized by Fluoro/phosphorimager Typhoon Trio (GE Healthcare).

### Statistical analyses

Statistical analysis of experimental data was done using the ordinary two-way ANOVA tests and multiple comparison was done using uncorrected Fisher's LSD tests.

### 3D images

Images were created using PyMOL Molecular Graphics System (Schrödinger, Inc.).

## Results

### Modification enzymes alleviate the Δ10 strain growth defect

We constructed an *E. coli* strain lacking 10 rRNA MEs (*ΔrlmB*, *ΔrlmE, ΔrlmKL*, *ΔrlmM*, *ΔrlmN*, *ΔrluB*, *ΔrluC*, *ΔrluE*, *ΔrluF* and *ΔrlhA*), resulting in the absence of all MNs (except for dihydrouridine at position 2449, for which the corresponding ME gene is unknown) within a 25 Å radius of the PTC (Table 1 and Figure 1). The absence of corresponding MNs in rRNA was confirmed by nucleoside analysis using RP-HPLC (Supplementary Figure S1). For comparison, the *Δ9* strain, which has the same ME knocked out as the Δ*10* strain, except for the chromosomal *rlmE* gene responsible for methylation of Um2552, was constructed.

**Figure 2.**
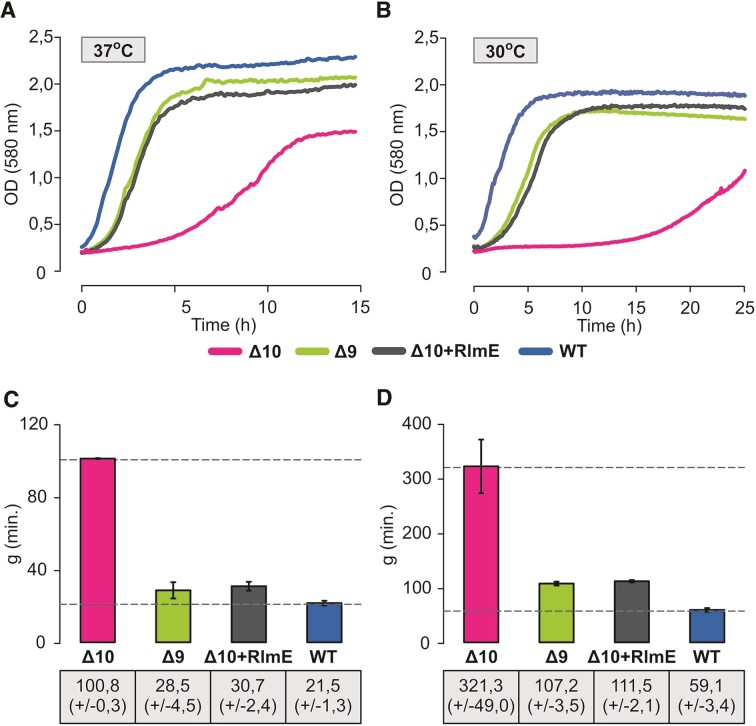
Growth curves and doubling times of multi-modification enzyme deletion strains Δ10 and Δ9. *E. coli* WT (MG1655), multi-ME deletion strains Δ9 and Δ10, as well as the Δ10 strain expressing plasmid-borne RlmE (Δ10 + RlmE), were grown in rich medium at 37°C or 30°C. OD_580_ was determined every 7 min, and growth curves were plotted. Panel **A** shows representative growth curves at 37°C and panel **B** at 30°C. Average doubling times (g) and standard deviations were calculated from at least three biological replicates with at least three technical replicates each at 37°C (panel **C)** and 30°C (panel **D**). WT and Δ10 average g values are shown in dotted lines for reference (C and D).

**Figure 3.**
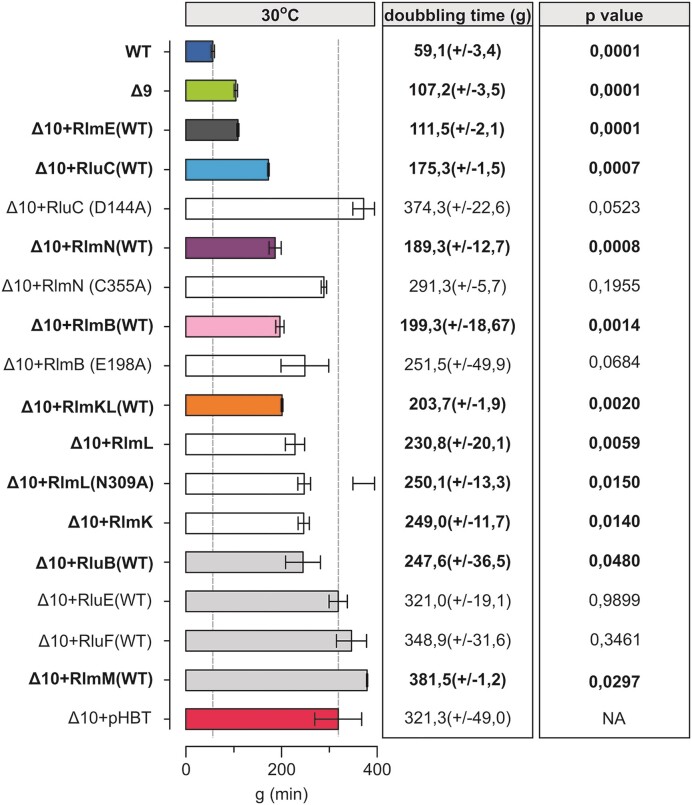
Effect of the expression of PTC modification enzymes on Δ10 strain doubling times at 30°C. *E. coli* WT (MG1655), Δ10 + pHBT and Δ10 strains expressing plasmid-borne native or mutated PTC MEs were grown in LB medium at 30°C. Average doubling times (g) and standard deviations were calculated as above. Δ10 + pHBT is the Δ10 strain with an ‘empty’ plasmid. *P*-values in respect of Δ10 strain are shown.

As expected, the *Δ10* deletion strain exhibits a severe growth defect, as revealed by 4.7 times and 5.4 times longer doubling times at 37°C and 30°C, respectively (Figure 2 and Figure S2A). The plateau was reached at a significantly lower cell density compared to the *WT E. coli* strain in rich medium at 37°C (Figure 2A). Expression of RlmE from a plasmid (*Δ10 + RlmE*) or genome (in the *Δ9* strain) improves growth substantially at both temperatures (Figure 2 and Figure S2A). The finding that both the *Δ9* strain and the Δ10 strain expressing plasmid-encoded RlmE protein (Δ10 + RlmE) exhibit only a minor growth defect at 37°C compared to the *WT* strain (Figure 2) suggests that *Δ10* growth defect is predominantly attributed to the lack of the RlmE protein. This agrees with the previous reports of the *rlmE* gene single knockout (*ΔrlmE*) causing major growth and ribosome assembly defects in *E. coli* (15–18). Notably, both *Δ9* and *Δ10 + RlmE* strains exhibit stronger growth defects at 30°C as compared to 37°C, indicating a cold-sensitive growth phenotype (compare Figure 3 and Figure S2). Simultaneous deletion of RluC and RlmE (ΔrluC/ΔrlmE) was earlier shown to cause a cold-sensitive growth phenotype (27). Since the *Δ9* strain also exhibits cold sensitivity (Figure 2), the cold-sensitive phenotype is not restricted to the absence of RlmE.

We then asked how individual MEs contribute to cell fitness. For that, we re-introduced them individually on a plasmid (or, in the case of RlmE, compared the *Δ10* and *Δ9*, and *Δ10 + RlmE* strains) and determined the growth at 37°C and 30°C. Re-introducing these MEs individually at a moderate expression level (leaky expression from pHBT-based vectors) restored the corresponding modifications (Figure S3) indicating that expression level of the MEs was sufficient for their enzymatic functions. Expression of the MEs rescued to varying extent the *Δ10* growth defect at both 30°C and 37°C (Figure 3 and Supplementary Figure S2A). Notable growth complementation of the *Δ10* strain was observed when re-introducing the native RluC protein that synthesizes three Ψs in 23S rRNA, two of which (Ψ2504 and Ψ2580) are in the PTC region, whereas the RluC aspartic acid-144 to alanine mutant RluC(D144A) further exacerbates the *Δ10* growth phenotype (Figure 3 and Supplementary Figure S2B). Aspartic acid residue 144 has previously been confirmed to be catalytically essential for pseudouridine formation (47). In agreement, HPLC analysis of rRNA nucleoside composition reveals an increase of Ψ’s in Δ10 rRNA from three to six when expressing native RluC but not the RluC(D144A) variant (Supplementary Figure S3). 23S rRNA of *WT* strain has 9 V. Hence, some or all of the three Ψ’s synthesized by the RluC protein contribute to bacterial fitness. The same can be said about the m^2^A2503 synthesized by RlmN, since the native but not the catalytically inactive RlmN(C355A) variant can alleviate the *Δ10* growth defect (Figure 3 and Supplementary Figure S2A and B). Cysteine 355 is known to be a crucial residue in RlmN-mediated RNA methylation (48,49), as also confirmed by our nucleoside composition analysis (Supplementary Figure S3). Since *E. coli* RlmN is not only responsible for the synthesis of m^2^A2503 in 23S rRNA but also for m^2^A synthesis at position 37 in a set (6) of tRNAs (12), the notable benefit to bacterial fitness could be attributed to either or both.

*E. coli* Gm2251 residue methylated by RlmB is located in the 23S rRNA P-loop, a universal site of post-transcriptional modification. The *E. coli ΔrlmB* single deletion strain can successfully compete with the *WT* strain and does not exhibit ribosome assembly defects (50). Expression of RlmB stimulates growth at both 37°C and 30°C in the *Δ10* strain (Figure 3 and Figure S2A). It appears that, in the absence of other PTC MEs, RlmB is important. Based on sequence conservation, RlmB crystal structure, and similarity to Pet56p, glutamic acid-198 has been predicted to play a crucial role in RlmB methyltransferase activity (50–52). The catalytically inactive variant of RlmB(E198A) that does not support methylation (Supplementary Figure S3) was able to stimulate the bacterial growth of the *Δ10* strain at 30°C in a plausible way (Figure 3 and Supplementary Figure S2B). Thus, *E. coli* RlmB has a second function independent of its methylase activity.

As mentioned (Table 1), *E. coli* RlmKL is a fused protein responsible for two MN in the PTC region. The m^2^G2445 and m^7^G2069 modifications are synthesized by its L- and K-domains, respectively (11,53). Introducing native RlmKL into *Δ10* alleviated moderately the growth defect at 30°C (Figure 3). The L- and K-domains of RlmKL can function as individual enzymes (53) (Supplementary Figure S3). Expression of the asparagine-309 to alanine mutant RlmL (RlmL(N309A)) abolishes the synthesis of m^2^G2445 (Supplementary Figure S3). Re-introduction of RlmK, RlmL, or RlmL(N309A) stimulates bacterial growth a little at 30°C, but does not have an effect at 37°C (Figure 3 and Supplementary Figure S2C). These results suggest a possible second function for RlmKL.

Expression of RlmM, RluB, RluE or RluF in the *Δ10* strain did not have a significant effect on the growth at 30°C or at 37°C (Figure 3 and Supplementary Figure S2A). Effect of RlhA was not analyzed in this study as this enzyme is important under oxidative stress and not under conditions used in this study (54).

Taken together, the *in vivo* complementation assay of the Δ*10* strain by MEs reveals that several PTC MEs (RlmB, RlmKL, RlmN, and RluC) can alleviate the growth defect of the *Δ10* strain (Figure 3 and Supplementary Figure S2A). Notably, RlmB and RlmL appear to have methylase-independent function(s), while either one or more pseudouridines made by RluC and m^2^A2503 (RlmN) are important as modifications. Hence, the significance of PTC MN for bacterial fitness is more complex and goes beyond what would be predicted solely based on their individual knockout studies. Therefore, a more detailed analysis of their contribution to ribosome biogenesis and function is called for. The interesting conclusion should be: some enzymes restore growth, likely because they modify rRNA (RluC, RlmN), and some restore growth even though they are catalytically inactive (RlmB, RlmKL). In the following studies, we examined ribosome assembly in the same set of strains.

### Lack of 23S rRNA modification enzymes leads to ribosome assembly defects

Modifications in the domain V of 23S rRNA are introduced at the early and intermediate stages of 50S subunit assembly, except for the late assembly-specific Um2552 (RlmE) and possibly ho^5^C2501 (14,54). It was proposed, therefore, that some of these MNs may play a role in ribosome biogenesis. However, until now, only RlmE has been shown to stimulate assembly of the 50S subunit (20–23,26,55–58).

**Figure 4.**
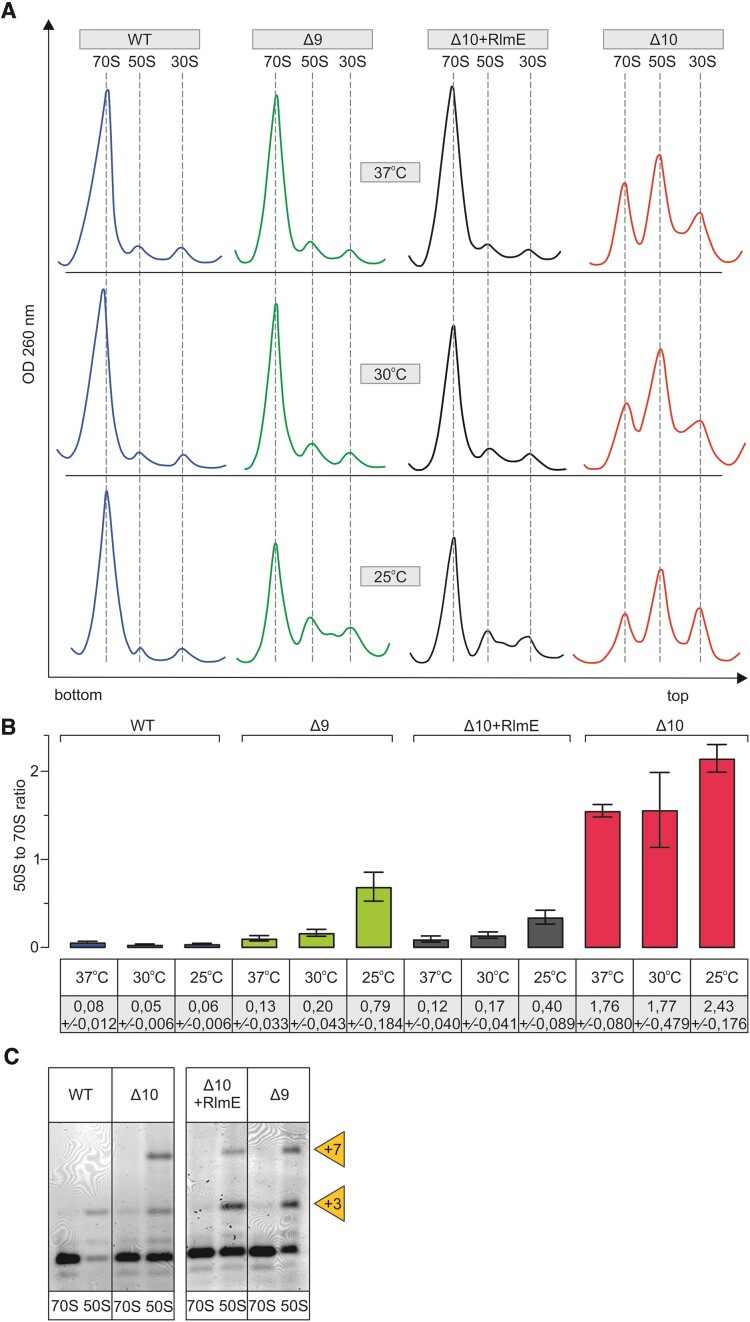
Ribosome biogenesis phenotype of multi-modification enzyme deletion strains Δ10 and Δ9. *E. coli* WT (MG1655), multi-ME deletion strains Δ9 and Δ10, as well as Δ10 strain expressing plasmid-borne RlmE (Δ10 + RlmE), were grown into early log phase (OD_600_ 0.4 - 0.5) at 37°C, 30°C and 25°C. Cells were lysed, and ribosome profiles were analyzed by sucrose gradient ultracentrifugation. Representative ribosome profiles of at least three independent samples are shown in panel **A**. The locations of fractions corresponding to 70S, 50S, and 30S are shown by dashed lines. Peak areas corresponding to 70S ribosomes and free 50S subunits were quantified, and 50S to 70S ratios are shown in panel **B**. At least two biological replicates with two technical replicates were used for calculations, with standard deviations shown. RNA was extracted from 70S and 50S fractions, and 23S rRNA 5′ends were mapped by primer extension analysis (panel **C**) using fluorescently labeled primer. cDNA fragments were resolved in a 7% polyacrylamide-urea gel, and fluorescence was visualized. +3 and + 7 refer to extra nucleotides at the 23S rRNA 5′ end.

We analyzed ribosome assembly in the modification-deficient strains at 37°C, 30°C or 25°C using sucrose density gradient centrifugation. In addition, the processing of the 5′end of 23S rRNA, the r-protein content of ribosomal particles, and low [Mg^2+^]-induced alterations were determined. The parent MG1655 strain (*WT*) exhibited a similar ribosome particle profile at all temperatures, where 70S particles constitute >90% of all ribosomal particles (Figure 4A, B). In the Δ10 strain, excesses of the free 30S and 50S particles accumulate at the cost of 70S (Figure 4A-B). This effect is stronger at lower temperatures (Figure 4A-B). RlmE complementation from a plasmid (*Δ10 + RlmE*) or genome (in the *Δ9* strain) restores WT-like ribosome profiles at 37°C in the early-log phase (Figure 4A) as well as in the mid-log phase (Supplementary Figure S4). The accumulation of free 50S and slower sedimenting particles occurs in both strains (*Δ10 + RlmE* and *Δ9*) in both growth phases at 25°C (Figure 4A and Supplementary Figure S4). These results clearly demonstrate that RlmE has a function in ribosome assembly, in agreement with the earlier observations (20–23,26,55–58). Moreover, in contrast to the previous studies, our new data show that RlmE is able to rescue ribosome assembly defects in the absence of other PTC MEs, demonstrating its utmost importance. However, expression of RlmE does not abolish the ribosome assembly defect of the Δ10 strain completely, in particular at 25°C.

The rate-limiting step of large ribosome subunit assembly is the late stage, when subunits have acquired their sedimentation rate value of 50S (38,40). Therefore, the 50S gradient peak most likely represents free subunits unable to associate with the 30S subunits due to incomplete subunit assembly. To test whether these free subunits are indeed ribosome assembly intermediates, we have analyzed the processing status of the 23S rRNA 5′ end, as it is a good indicator of the 50S assembly status. The mature ends of 23S rRNA are formed exclusively at the stage of 70S ribosomes (59,60). Mapping of the 5′ end reveals that the 23S rRNA of free 50S particles in the *WT* strain and in the *Δ10*, *Δ9* and *Δ10 + RlmE* strains is extended by three (+3) or seven (+7) nucleotides with respect to the mature 5′end. (+3) and (+7) ends of pre-23S rRNA correspond to RNase III cleavage sites (Figure 4C (61)). RNase III is the first rRNA processing enzyme that liberates 16S and 23S rRNA precursors from the primary 30S transcript (62). Thus, 23S rRNA is incompletely processed, demonstrating that the free 50S subunits of *Δ10*, *Δ9* and *Δ10 + RlmE* strains are ribosome assembly intermediate particles. Another indicator of the completeness of the ribosome large subunit assembly is its sedimentation rate at 0.5 mM [Mg^2+^] (23,58). When the free 50S particles were isolated in the presence of 10 mM [Mg^2+^] and subsequently analyzed by centrifugation in the presence of 0.5 mM [Mg^2+^], 45S particles appeared both in the *Δ10* and *Δ9* strains but not in the *WT* strain (Supplementary Figure S5). The free LSU particles of the *Δ10* strain are divided into 45S (major) and 50S (minor) peaks at 0.5 mM [Mg^2+^] (Supplementary Figure S5). Free 50S ribosome subunits of the *Δ9* strain are divided into 45S (minor) and 50S (major) particles at 0.5 mM [Mg^2+^] (Supplementary Figure S5). Previous reports show that immature LSU from the *ΔrlmE* strain also migrate as 45S particles at 0.5 mM [Mg^2+^], but when re-run at a higher [Mg^2+^] concentration, they migrate at the 50S position and are genuine precursors of the 50S subunit rather than dead-end products (23,58). Hence, immature 50S particles of the strains *Δ10* and *Δ9* are also likely precursors of mature 50S subunits. The findings demonstrate that the formation of 45S particles at 0.5 mM [Mg^2+^] is not specific to RlmE-deficient strains but is induced by the absence of other ME(s) as well. Ribosome large subunit assembly status was further characterized by the r-protein composition of the assembly intermediate particles (Supplementary Figure S6). Free 50S particles were compared with stable isotope-labelled standard 70S of the *WT* strain (see Materials and Methods) using quantitative LC-MS/MS (63). Overall, only minor differences in r-protein content were observed in the free 50S subunits of *Δ9* and *Δ10* strains (Supplementary Figure S6). Thus, r-protein deficiency cannot account for the major ribosome assembly defect. Slightly reduced levels of uL16 and bL35 in the free LSU particles of *Δ10*, *Δ9* and *Δ10 + RlmE* strains imply that they are halted in the late assembly stages.

### Modification enzymes stimulate large ribosome subunit assembly

**Figure 5.**
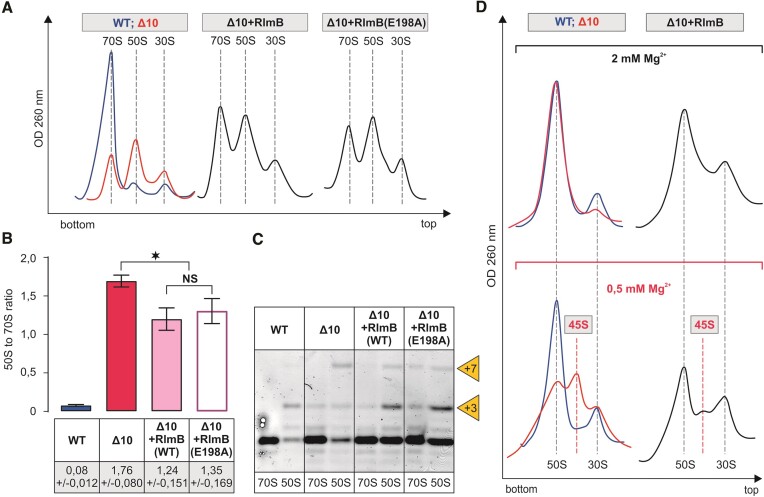
Effect of RlmB on the ribosome biogenesis phenotype of the Δ10 stain. *E. coli* WT (MG1655), Δ10, as well as Δ10 strains expressing plasmid-borne native or mutant RlmB, were grown into late log phase (OD_600_ ∼ 1.5) at 37°C. Cells were lysed, and ribosome profiles were analyzed by sucrose gradient ultracentrifugation. Representative ribosome profiles of at least three independent samples are shown in panel **A**. The locations of fractions corresponding to 70S, 50S and 30S are shown by dashed lines. Peak areas corresponding to 70S ribosomes and free 50S subunits were quantified, and 50S to 70S ratios are shown in panel **B**. At least two biological replicates with two technical replicates were used for calculations, with standard deviations shown. RNA was extracted from 70S and 50S fractions, and 23S rRNA 5′ends were mapped by primer extension analysis. cDNA fragments were resolved in a 7% polyacrylamide-urea gel, and fluorescence was visualized (panel **C**). +3 and + 7 refer to extra nucleotides at the 23S rRNA 5′ end. Sedimentation coefficients of *in vivo* 50S assembly intermediates from WT, Δ10, and Δ10 strains expressing RlmB under different Mg^2+^ concentrations (panel **D**). Free 50S subunit fractions from sucrose density gradient centrifugation (in the presence of 10 mM Mg^2+^) were analyzed further by 10% to 25% sucrose density gradient centrifugation in the presence of 2 mM or 0.5 mM Mg^2+^ (Materials and Methods). Ribosome profiles were recorded at OD_260_, and representative profiles from at least two repeats are shown. 45S approximates the sedimentation coefficient for intermediate-size particles. Note that 30S particles are contaminating 50S particles, and their fraction does not depend upon Mg^2+^ concentration.

In order to detect changes in the ribosome assembly induced by the re-introduction of MEs into the Δ10 strain, the ribosome profile was analyzed in the late log growth phase cells (OD_600_ ∼ 1.5). Expression of RlmB in the strain *Δ10* restores Gm2251 (Supplementary Figure S3) and leads to an increase in the 70S and a corresponding decrease in the free 50S particles according to the sucrose gradient profile in the late exponential phase culture (Figure 5A, B) when rRNA synthesis is low and the accumulation of free subunits indicates an assembly defect of the rRNA molecules made several minutes earlier. Thus, RlmB appears to help ribosome 50S assembly at 37°C the *Δ10* strain (Figure 5). In the *Δ10 + RlmB* strain, the fraction of 45S particles at 0.5 mM [Mg^2+^] is minor (Figure 5), as compared to the *Δ10* strain and even to the *Δ9* strain (Supplementary Figure S5). This result indicates that RlmB significantly improves the compactness of 50S particles. It appears that the MNs around the PTC have redundant functions in ribosome assembly; the role of RlmE can be partially complemented by RlmB. Importantly, the catalytically inactive RlmB(E198A) stimulates ribosome biogenesis to nearly the same extent as the native RlmB (Figure 5A-B). The major processing product at the 23S rRNA 5′ end is +3 in both *Δ10 + RlmB* and *Δ10 + RlmB(E198A)* (Figure 5C). Thus, RlmB appears to have a function in ribosome LSU assembly that is distinct from its methylase activity.

**Figure 6.**
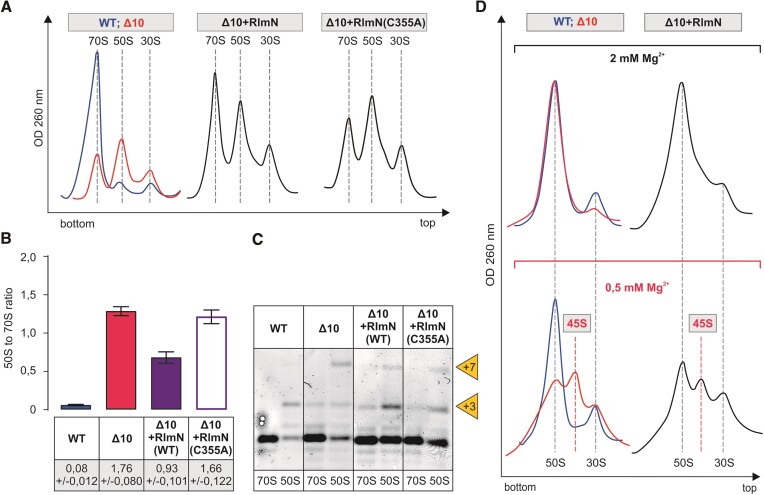
Effect of RlmN on the ribosome biogenesis phenotype of the Δ10 stain. *E. coli* WT (MG1655), Δ10, as well as Δ10 strains expressing plasmid-borne native or mutant RlmN, were grown into late log phase (OD_600_ ∼1.5) at 37°C and were analyzed as described in the legend of Figure 5. Representative ribosome profiles of at least three independent samples are shown in panel **A**. Peak areas corresponding to 70S ribosomes and free 50S subunits were quantified, and 50S to 70S ratios are shown in panel **B**. 5′ends of rRNA from the 70S and 50S fractions were mapped by primer extension analysis. (panel **C**). +3 and + 7 refer to extra nucleotides at the 23S rRNA 5′ end. Sedimentation coefficients of *in vivo* 50S assembly intermediates from WT, Δ10 and Δ10 strains expressing RlmB under different Mg^2+^ concentrations (panel **D**). Free 50S subunit fractions from sucrose density gradient centrifugation (in the presence of 10 mM Mg^2+^) were analyzed further by 10% to 25% sucrose density gradient centrifugation in the presence of 2 mM or 0.5 mM Mg^2+^ (Materials and methods). Ribosome profiles were recorded at OD_260_, and representative profiles from at least two repeats are shown. 45S approximates the sedimentation coefficient for intermediate-size particles. Note that 30S particles are contaminating 50S particles, and their fraction does not depend upon Mg^2+^ concentration.

The ribosome assembly phenotype of the RlmN (m^2^A2503) deletion strain (ΔrlmN) has not been previously reported. The effect of RlmN expression in the *Δ10* strain on the 50S assembly is reminiscent of RlmB according to sucrose gradient centrifugation and 23S rRNA processing status (Figure 6). RlmN restores m^2^A2503 in the *Δ10* strain (Supplementary Figure S3). Ribosome assembly in the late exponential phase *Δ10 + RlmN* strain cells is restored significantly as compared to the Δ10 strain, as evident from the major 70S and small 50S peaks on the sucrose gradient profile and the reduction of the 45S fraction at 0.5 mM [Mg^2+^] (Figure 6). The catalytically inactive RlmN(C355A) variant, however, does not stimulate ribosome assembly according to the ribosome sucrose gradient profile (Figure 6). It appears that the methylation of A2503 is important for large ribosome subunit assembly.

**Figure 7.**
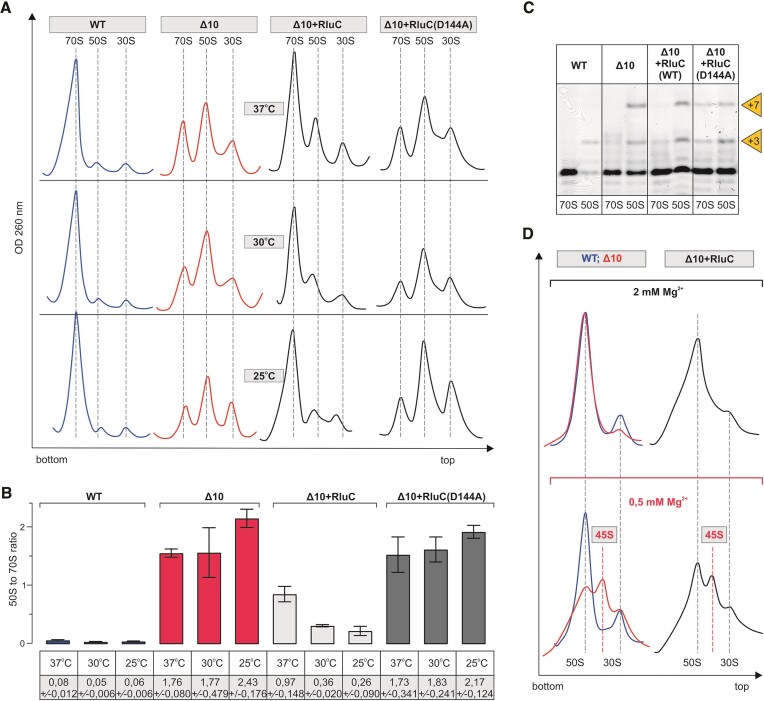
Effect of RluC on the ribosome biogenesis phenotype of the Δ10 stain at different temperatures. *E. coli* WT (MG1655), Δ10, as well as Δ10 strain expressing plasmid-borne native or mutant RluC were grown at 37, 30, and 25°C. Cells were collected at late log phase (OD_600_ ∼ 1.5), lysed and ribosome profiles were analyzed by sucrose gradient ultracentrifugation. Representative ribosome profiles of at least three independent samples are shown. The locations of fractions corresponding to 70S, 50S and 30S are shown by dashed lines. Peak areas corresponding to 70S ribosomes and free 50S subunits were quantified, and 50S to 70S ratios are shown. At least two biological replicates with two technical replicates were used for calculations, with standard deviations shown. RNA was extracted from 70S and 50S fractions, and 23S rRNA 5′ends were mapped by primer extension analysis using fluorescently labeled primer. cDNA fragments were resolved in a 7% polyacrylamide-urea gel and fluorescence was visualized. +3 and +7 refer to extra nucleotides at the 23S rRNA 5′ end. Free 50S subunit fractions from sucrose density gradient centrifugation (in the presence of 10 mM Mg^2+^) were analyzed further by 10–25% sucrose density gradient centrifugation in the presence of 2 mM or 0.5 mM Mg^2+^ (Materials and methods). Ribosome profiles were recorded at an OD of 260 nm. 45S approximates the sedimentation coefficient for intermediate-size particles and 30S subunit is an impurity.

Using sucrose gradient centrifugation, the impact of RluC on ribosome assembly was analyzed in the *Δ10* strain at 37°C, 30°C and 25°C. The ribosome assembly defect of the Δ10 strain is alleviated by RluC at all temperatures, as demonstrated by major 70S and minor 50S peaks and the incompletely processed 5′end of 23S rRNA (Figure 7). Interestingly, expression of RluC increases the fraction of 70S in the cells more at lower temperatures and thereby helps ribosome assembly in a temperature-dependent manner. This phenomenon can partially explain the cold-sensitive phenotype of the RlmE/RluC double deletion strain (27). When isolated free 50S particles of the *Δ10 + RluC* strain are analyzed at 0.5 mM [Mg^2+^], the proportion of the 50S fraction increases and the 45S fraction decreases as compared to the corresponding *Δ10* particles (Figure 7), demonstrating stimulation of 50S subunit assembly. Expression of the catalytically inactive variant of RluC(D144A) does not improve ribosome assembly in the Δ10 strain (Figure 7), suggesting that one or more pseudouridines made by RluC are important for ribosome LSU assembly.

The *ΔrlmKL* single knockout strain exhibited no significant change in ribosome profile (53). However, in the *Δ10* background, the most notable improvement on the ribosome biogenesis phenotype was observed with expressing the fused RlmKL protein, as demonstrated by major 70S and small 50S peaks. Notably, the 50S to 70S ratio of native and catalytically inactive L-domains (RlmL and RlmL(N309A), respectively) is very similar (Supplementary Figure S7). Expressing native K-domain (RlmK) does not have a notable effect on the ribosome assembly (Supplementary Figure S7). Thus, it appears the RlmL domain of RlmKL rather than the corresponding m^2^G2445 plays a role in the large ribosome subunit assembly.

Expression of RlmM, RluB, RluE and RluF does not have a significant effect on the sucrose gradient profile or to the 50S to 70S ratio in the Δ10 strain (Supplementary Figure S8) suggesting that these enzymes do not rescue ribosome assembly defects. It does not exclude their involvement in the ribosome assembly process, but it demonstrates that these enzymes do not sufficiently accelerate ribosome production to be detected by our approach.

Taking together, the assembly effects of the PTC modification enzymes, it is evident that RlmB, RlmN, RluC and RlmL have functions during ribosome 50S assembly in addition to the previously known RlmE. In the cases of RlmN and RluC, the modifications appear to be important. However, RlmB has a seemingly modification-independent function as well. In the case of the fused RlmKL protein, its RlmL domain rather than the corresponding m^2^G2445 plays a role in ribosome assembly, possibly via its RNA helicase activity.

## Discussion

Functional ribosome structure is formed during the ribosome assembly process, which is directed by ribosomal and non-ribosomal proteins (42). In *WT E. coli* cells, the majority of the r-proteins bind to the rRNA, and the final sedimentation value of the subunits is acquired quickly, during less than 1 minute after transcription (38). When an assembly factor is absent or insufficient r-proteins are available, ribosome assembly is slowed down, leading to the accumulation of intermediate particles with a lower S value (39,64). Moreover, the absence of different assembly factors leads to alternative assembly pathways or assembly landscapes, as demonstrated by cryogenic EM studies (39). rRNA modifications around the PTC are made during the early or intermediate stages of ribosome LSU assembly (15) (14) when late assembly proteins are not bound (64). RlmE and probably RlhA act during the late stages of LSU assembly (14,15,54). The question whether or not rRNA modification affects ribosome subunit assembly in bacteria is poorly understood. Until now, only RlmE has been shown to stimulate the assembly of the 50S subunit (20–23,26,55–58).

Functional 50S particles can be assembled without 23S rRNA modifications, as demonstrated by using *in vitro* transcribed *E. coli* 23S rRNA (modification-free 23S rRNA) able to catalyze peptide bond formation (65). On the other hand, the *ΔrluC* (10,66), *ΔrlmN* (67), *ΔrlmB* (50), and *ΔrlmKL* (53,68) single knockout strains, as well as the Δ5 (*rluC*, *rlmKL*, *rlmN*, *rlmM* and *rluE* knockout) (27) and the Δ7 strain lacking all pseudouridines in the rRNA (*rsuA*, *rluA*, *rluB*, *rluC*, *rluD*, *rluE* and *rluF* knockout) (28) multi-deletion strains have very small growth defects compared to the *WT* strain. The *E. coli* strain *Δ10* lacking 10 genes encoding enzymes responsible for modification of 23S rRNA around the PTC, is viable. This result demonstrates that the ribosomes lacking rRNA MNs around the PTC are able to carry out protein synthesis *in vivo*. However, *Δ10* strain growth is impaired, in particular at 30°C. A cold-sensitive growth phenotype has been observed for the strain lacking just two MEs, RlmE and RluC [8]. The results obtained in this work demonstrate that the Δ9 strain, where RlmE is present, is cold-sensitive as well. Thus, cold sensitivity is not restricted to RlmE but seems to be a more general phenomenon associated with the rRNA modification pattern. Cold sensitivity is often observed in strains where ribosome assembly is defective (42). The results above establish the functional roles of RlmB, RlmKL, RlmN, and RluC in ribosome large subunit assembly.

Incompletely assembled large ribosome subunits accumulating in the *Δ10* strain have a nearly complete set of LSU proteins and incompletely processed 23S rRNA. The accumulating 50S particles are prone to low [Mg^2+^]-induced slow sedimentation. The sub-stoichiometric presence of r-proteins uL16 and bL35 (Supplementary Figure S6) in Δ10 free 50S particles agrees with the cryo-electron microscopy analyses of *ΔrlmE* strain 50S assembly intermediates that also revealed notable structural differences near PTC, such as H38, H69-71 and H89-93 (58). While we could not quantify bL36 it was reported to be absent from the 45S precursor in the *ΔrlmE* strain (23). Arai et al. proposed that bL36 incorporation, in concert with the Um2552-mediated stabilization of association between 23S rRNA H92 and H71, triggers late steps of 50S subunit assembly (23). The findings that the *ΔrlmE* slow growth phenotype can be alleviated by overexpressing the assembly factors small GTPases EngA and ObgE (26) demonstrate that RlmE has an activity that is independent of Um2552 synthesis. Moreover, Spb1, which is responsible for 2′-O methylation of U in the A loop of the yeast ribosome, is an essential protein but not its product, Um2921 (69). Thus, the second function of Um2552 methylases seems to be a conserved feature from bacteria to Eukarya. On the other hand, the *E. coli* 23S rRNA U2552C mutant strain also reveals the accumulation of 50S precursor particles (23). Depletion of SAM was implicated in affecting ribosome assembly through hypomodification of Um2552 (57). Taken together, both Um2552 and methylation-independent functions are likely important for ribosome LSU assembly. Previously, *in vitro* kinetic studies have shown that while the 70S ribosomes of the *ΔrlmE* strain show no defect in peptide bond formation, peptide release, or ribosome recycling at 37°C, they translocate 20% slower than *WT* ribosomes during each round of elongation, which, together with affected EF-G turnover, slows down the overall rate of translation (58).

Expression of RlmB appears to help ribosome 50S assembly in the *Δ10* strain (Figure 5A). RlmB (Gm2251) ortholog Pet56p has an essential role in the maturation of the yeast *Saccharomyces cerevisiae* mitochondrial large ribosome subunit, independent of its methyltransferase activity (52). Notably, *Saccharomyces cerevisiae* mitochondrial LSU rRNA has only three MNs. However, since the *ΔrlmB* single knockout did not show any ribosome assembly defect or growth phenotype, it was concluded that RlmB has no important role in ribosome assembly or function in *E. coli* (50). Expression of RlmB in the *Δ10* strain facilitates ribosome LSU assembly and improves the compactness of the free 50S subunits (Figure 5). It appears that the MNs around the PTC have redundant functions in ribosome assembly; the role of RlmE can be partially complemented by RlmB during ribosome LSU assembly. Considering that the catalytically inactive RlmB variant RlmB(E198A) can stimulate 50S assembly (Figure 5), its function is reminiscent of that of the RlmE, as both appear to have dual roles in ribosome large subunit assembly: 2′-O methylation and a methylation-independent role. Omission of one out of three MNs in yeast mt LSU rRNAs has a significant effect on ribosome assembly [22]. Thus, numbers matter for modifications in ribosome assembly.

Deletion of the *rluC* gene (*ΔrluC)* does not appear to affect ribosome assembly (70). Furthermore, as discussed previously, deletion of all seven rRNA-specific pseudouridine synthases has only a marginal effect on ribosome assembly (28). However, RluC does seem to have a possible link to ribosome assembly through the elongation factor family GTPase BipA. Namely, deletion of *rluC* suppresses the ribosome assembly defect caused by the deletion of *bipA* at low temperatures, suggesting that ribosomes unmodified by RluC do not depend on BipA for efficient assembly (70). The data in Figure 7 demonstrate that RluC, but not its catalytically inactive variant, stimulates large ribosome subunit assembly, in particular at temperatures below 37°C. The temperature effect on the stimulation of ribosome assembly is opposite when RluC and RlmE are compared. RluC stimulates ribosome assembly more at lower temperatures and RlmE at higher temperatures (Figures 7 and 4, respectively). In this context, it is interesting to remember that RlmE was first identified as a heat shock protein (FtsJ) affecting cell division (71). Stimulation of ribosome LSU assembly by RluC in the *Δ10* strain reveals that RluC can complement RlmE during ribosome assembly.

Native but not methylase-inactive variant of RlmN stimulates large ribosome subunit assembly (Figure 6). m^2^A is located at the entrance of the nascent peptide exit tunnel, and it has been proposed that it relay specific nascent chain stalling signals to the PTC (72). It should be noted that RlmN is a dual-specificity enzyme that is also responsible for m^2^A synthesis at purine 37 in a set of tRNAs (12), and it cannot be excluded that the phenotypes associated with RlmN could also be due to tRNA modification. However, the error-prone (increased misreading of the UAG stop codon) translation phenotype observed in the *ΔrlmN* strain is believed to be due to the loss of m^2^A in 23S rRNA rather than in tRNA (12). Accordingly, m^2^A2503 has been linked to translational proofreading at PTC (12).

Kimura et al. (53) have reported a Helix 74 unwinding activity for RlmKL that facilitates the cooperative synthesis of m^7^G2069 and m^2^G2445. This could also underlie the involvement of RlmKL in stimulating the assembly of 50S subunits in a strain lacking the RNA helicase DeaD (53). RlmKL was found to associate with the 45S assembly precursor particles of the *ΔrlmE* strain (23) suggesting involvement in LSU assembly. Interestingly, it has been reported that the N-terminal L-domain activity of RlmKL for m^2^G2445 formation is significantly enhanced by the C-terminal K-domain (53), and RlmK and RlmL methyltransferases exist as separated proteins in other bacteria (11). Only the L domain of RlmKL is sufficient to stimulate ribosome LSU assembly in the Δ10 strain (Supplementary Figure S7). Moreover, the methylase-inactive variant of RlmL(N309A) stimulates LSU assembly to the same degree as functional RlmL (Supplementary Figure S7A, B). Therefore, the assembly function of RlmL can be attributed to its RNA helicase activity.

Most 23S rRNA modifications around the PTC are specific to the early or intermediate stages of ribosome LSU assembly, as mentioned above. Yet, the absence of 10 MEs leads to the accumulation of 50S particles with a full complement of LSU proteins (Supplementary Figure S6). Such particles are late assembly-specific. Interestingly, domain V of 23S rRNA containing PTC modifications is the last assembly block both *in vivo* (39) and during *in vitro* reconstitution (40). It appears that PTC modifications are introduced before or during the folding of domain V of 23S rRNA. MEs can affect rRNA structure by recognizing specific folds and thereby directing ribosome assembly. The cold sensitivity conferred by the absence of MEs supports the idea that MEs reduce the activation energy of conformational transitions. We hypothesize that RlmB, RlmKL, RlmN and RluC stimulate folding of the 23S rRNA around the PTC either by binding to the RNA (RlmB) RNA helicase activity (RlmKL) or via corresponding modification (RlmN and RluC). The modification site of RlmB is in loop 80 (P loop). The P loop (from G2251 to G2253) can form a misfolded structure by base-pairing with a part of helix 89 (from C2498 to U2500) (73), which is an important part of PTC. RlmB can potentially avoid such a misfolding event, similar to what has been shown for 5S rRNA (73). Stimulation of the assembly of PTC around H89 and H91 is assisted by ribosomal proteins uL16, eL40 and assembly factors Lsg1 and Nmd3 in eukaryotes (74). In bacteria, uL16 is critical for activation of the PTC (75). MEs can play similar roles in bacteria as eukaryotic ribosome assembly factors. In the absence of MEs, this would lead to a nonoptimal 23S rRNA folding pathway, which in turn slows down ribosome LSU assembly and results in the accumulation of precursor particles. As RlmB, RlmKL, RlmN and RluC appear to function around the same region of 23S rRNA during the early stage of ribosome assembly, they can complement each other in the folding of 23S rRNA around PTC. Notably, RlmB, RlmE, RlmN and RluC all help to form compact 50S particles resistant to low [Mg^2+^]-induced perturbation. RlmKL, RluB and RluC, together with three RNA helicases (DeaD, RhlE, and SrmB), are found in the precursor particles accumulating in the absence of RlmE (23). The presence of these early assembly-specific factors in the late assembly particles may reflect that their rRNA folding job has not been completed. A final conclusion of the results obtained is that there is a mutual interdependence between 23S rRNA modification and ribosome LSU assembly. Modification of rRNA during specific assembly stages has been well documented by several studies. This study demonstrates that the opposite relation, progression of the ribosome assembly depends on the rRNA modification, applies to ribosome biogenesis in bacteria.

## Supplementary Material

gkae222_Supplemental_File

## Data Availability

Mass spectrometry data of ribosomal proteins can be found at EMBL-EBI PRoteomics IDEntification database (PRIDE). Dataset accession codes PXD047588 and PXD047376.

## Notes

Present address: Rya Ero, Nanyang Technological University, School of Biological Sciences, 59 Nanyang Drive, Singapore 636921.
