# Supplementary material for: Ribosomal RNA modification enzymes stimulate large ribosome subunit assembly in E. coli
Source: Nucleic Acids Res. 2024 Mar 30;52(11):6614–28. doi: 10.1093/nar/gkae222 (PMC11194073; doi:10.1093/nar/gkae222)
Supplement: gkae222_Supplemental_File [file gkae222_supplemental_file.pdf]

SUPPLEMENTARY DATA

Ribosomal RNA modification enzymes stimulate large ribosome subunit assembly in *E. coli*.

Rya Ero*, Margus Leppik, Kaspar Reier, Aivar Liiv and Jaanus Remme

Affiliations: IMCB University of Tartu, Riia 23, 51010 Tartu, Estonia

Correspondence to: J. Remme

E-mail: jremme@ut.ee

*Present address: Nanyang Technological University,

School of Biological Sciences, 59 Nanyang Drive, Singapore 636921

Supplementary methods

Nucleoside composition analysis

50S subunits were dissociated and purified by sucrose gradient from sucrose-gradient-purified 70S ribosomes. 70S ribosomes were pelleted by centrifugation ($\omega^2t=1.2\ x10^{12}$ rad/sec), suspended in OV buffer (20 mM Tris-HCl; 100 mM KCl; 1 mM $Mg(OAc)_2$; 6 mM 2-mercaptoethanol) and loaded onto a 10-25% sucrose gradient in the same buffer. Ribosome profiles were recorded following ultracentrifugation ($\omega^2t=2.7\ x10^{11}$ rad/sec) and 50S subunit fractions were collected. Mg^{2+} was adjusted to 10 mM and 50S pelleted by ultracentrifugation as previously described. 50S subunits were suspended in TEN buffer (100 mM NaCl; 10 mM Tris-HCl pH 7.5; 1 mM EDTA), flash-frozen in liquid nitrogen, and stored at -80°. Phenol-chloroform treatment followed by ethanol precipitation was used for rRNA extraction from ribosome particles.

The nucleoside composition was analyzed by HPLC as described in (1). In short, rRNAs extracted from 50S subunits were degraded by nuclease P1 (Sigma) and treated with bacterial alkaline phosphatase (Thermo Fisher Scientific). The samples were then run on a Supelcosil LC-18-S reverse-phase HPLC column, the nucleoside peaks were identified based on relative motilities, and peak areas were integrated.

Mass-spectrometry

Ribosome r-protein content was analyzed by mass spectrometry as described in (2). In short, free 50S ribosomes from MG1655, Δ9, Δ10, or Δ10 strains expressing RlmE were mixed in a 1:1 molar ratio with reference 70S from *E. coli* MG1655-SILAC (F− λ− rph-1 ΔlysA ΔargA) strain grown in MOPS medium (3)

supplemented with 0.1 mg/mL medium-heavy-labeled arginine (Arg6; $[^{13}C]_6H_{14}N_4O_2$) and lysine (Lys8; $C_6H_{10}[^2H]_4N_2O_2$) (Silantes). Proteins were dissolved in an 8 M urea/2 M thiourea solution, reduced for 1 h at 56°C by adding 1 mM dithiothreitol (DTT), and carbamidomethylated with 5 mM chloroacetamide for 1 h in the dark. Proteins were digested with endoproteinase Lys-C and peptides were further digested using mass spectrometry-grade trypsin. The enzymes were inactivated with trifluoroacetic acid. For LC-MS/MS analysis, peptides were desalted on self-made reverse-phase C18 stop-and-go-extraction tip (STAGEtip) columns and analyzed by LC-MS/MS using the LTQ Orbitrap XL mass spectrometer (Thermo Scientific) coupled with an Agilent 1200 nanoflow LC system via a nanoelectrospray ion source (Proxeon).

Data analysis was performed using Maxquant (v1.5.6.0) with default settings (4), except that the minimal peptide length for the specific and non-specific searches was five amino acids. Unique and razor peptides were used for quantification, main search peptide tolerance was 8 ppm, and variable modification was used for quantitation of oxidation (methionine). The peptide identification search was carried out against the *E. coli* K-12 MG1655 protein sequence database from UniprotKB (as of March 2021). The search results were filtered and transformed using Perseus (v1.6.14.0) (5). Each protein was quantified through the SILAC ratio L/M, comparing unlabeled (L) relative quantities against medium-heavy labeled (M) internal reference. Mass spectrometry data of ribosomal proteins can be found at EMBL-EBI PRoteomics IDEntification database (PRIDE). Dataset accession codes PXD047588 and PXD047376.

Reference

1. Gehrke, C.W. and Kuo, K.C. (1989) Ribonucleoside analysis by reversed-phase high-performance liquid chromatography. *J Chromatogr*, **471**, 3-36.
2. Reier, K., Lahtvee, P.J., Liiv, A. and Remme, J. (2022) A Conundrum of r-Protein Stability: Unbalanced Stoichiometry of r-Proteins during Stationary Phase in Escherichia coli. *mBio*, **13**, e0187322.
3. Neidhardt, F.C., Bloch, P.L. and Smith, D.F. (1974) Culture medium for enterobacteria. *J Bacteriol*, **119**, 736-747.
4. Cox, J. and Mann, M. (2008) MaxQuant enables high peptide identification rates, individualized p.p.b.-range mass accuracies and proteome-wide protein quantification. *Nature biotechnology*, **26**, 1367-1372.
5. Tyanova, S., Temu, T., Sinitcyn, P., Carlson, A., Hein, M.Y., Geiger, T., Mann, M. and Cox, J. (2016) The Perseus computational platform for comprehensive analysis of (prote)omics data. *Nature methods*, **13**, 731-740.

A

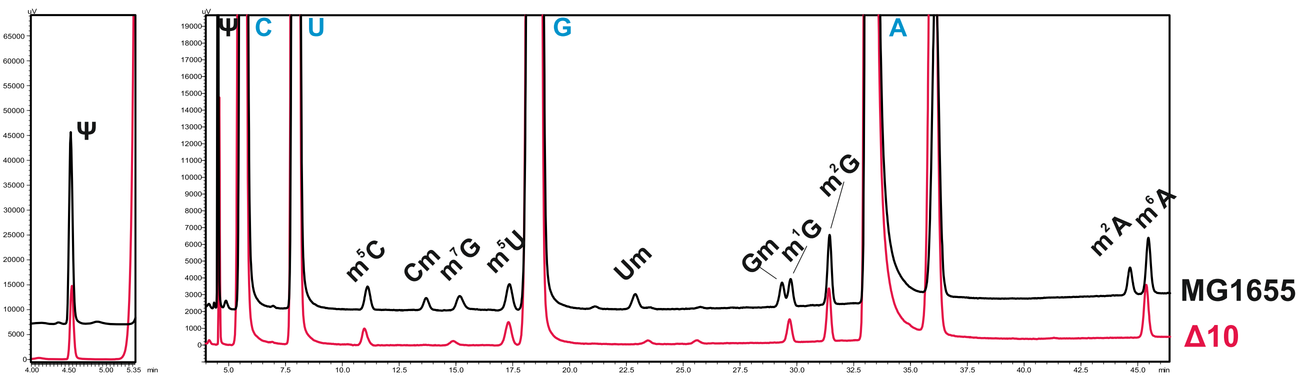

B

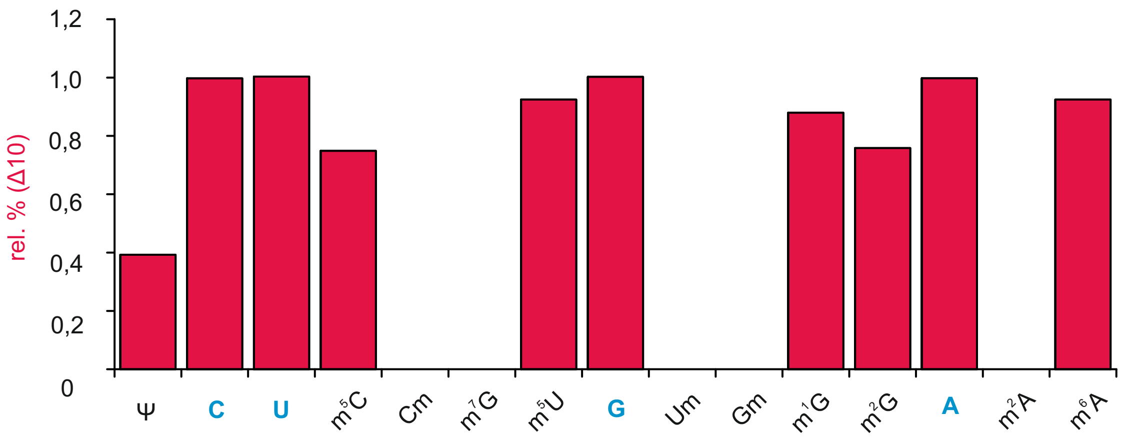

tot. MG1655 (23S)	9	636	537	1	1	1	2	907	1	1	1	2	759	1	2
tot. Δ10 (23S)	4	637	587	1	0	0	2	910	0	0	1	1	760	0	2
rel % Δ10 (23S)	44	100	101	100	0	0	100	100	0	0	100	50	100	0	100

Figure S1. Nucleoside composition of 23S rRNA of *WT* and *Δ0* strain.

50S subunits were purified from mature 70S ribosomes, rRNAs (23S and 5S) were extracted, digested with nuclease P1, and treated with alkaline phosphatase. Nucleosides were separated by high-performance liquid chromatography (HPLC) A_{260} was monitored and nucleosides were identified according to their retension times (see Supplementary Methods). Representative comparisons with WT are shown for *Δ10*. Modification peak areas were compared with WT controls normalized to 1. Quantifications of HPLC peak areas are included in Fig. 1B.

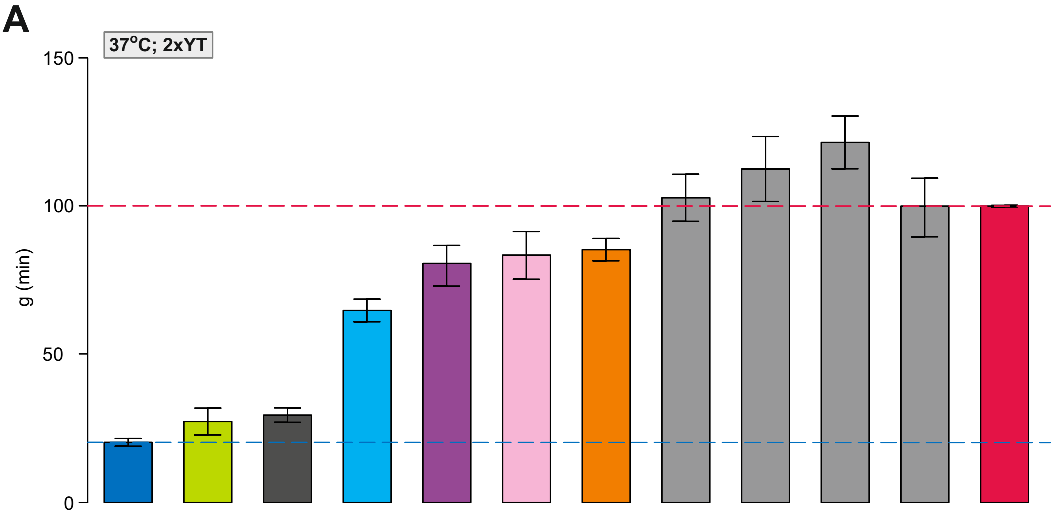

WT	Δ9	Δ10									
		RlmE	RluC	RlmN	RlmB	RlmKL	RluB	RluE	RluF	RlmM	pHBT
21,5 (+/-1,3)	28,5 (+/-4,5)	30,7 (+/-2,4)	65,7 (+/-3,8)	81,5 (+/-6,8)	84,3 (+/-8,0)	86,2 (+/-3,7)	103,6 (+/-7,9)	113,3 (+/-10,9)	122,2 (+/-8,9)	100,7 (+/-9,8)	100,8 (+/-0,3)

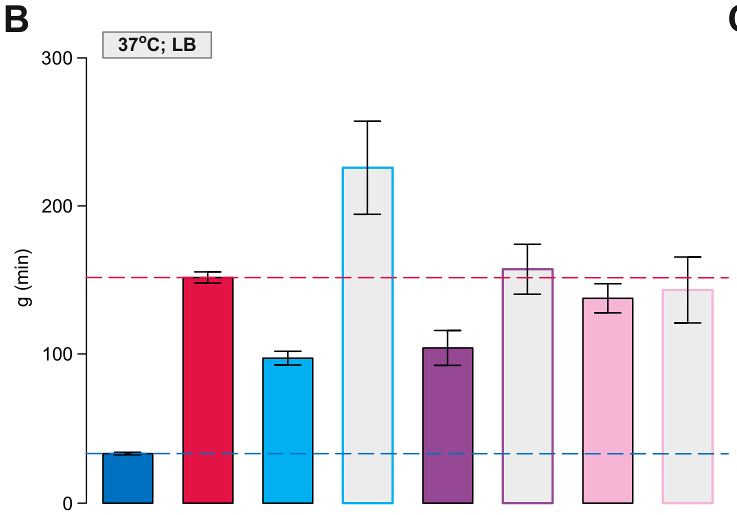

WT	Δ10						
	pHBT	RluC (WT)	RluC (D144A)	RlmN (WT)	RlmN (C355A)	RlmB (WT)	RlmB (198A)
35,9 (+/-0,9)	154,0 (+/-3,8)	100,0 (+/-4,6)	227,7 (+/-31,7)	106,9 (+/-11,8)	159,6 (+/-16,7)	140,2 (+/-9,7)	145,7 (+/-20,0)

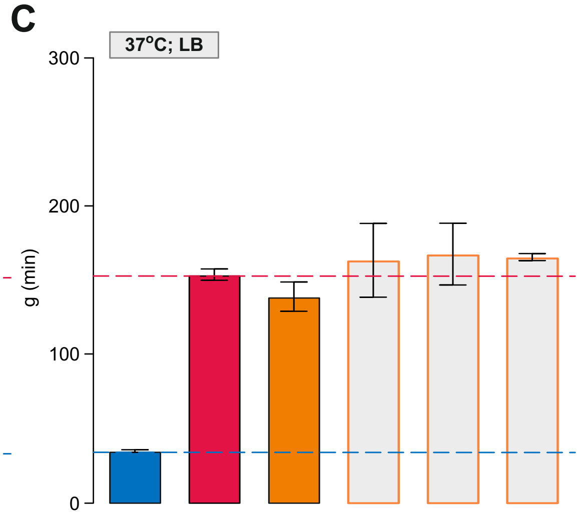

WT	Δ10				
	pHBT	RlmKL (WT)	RlmL (WT)	RlmL (N309A)	RlmK (WT)
35,9 (+/-0,9)	154,0 (+/-3,8)	139,3 (+/-9,8)	163,7 (+/-24,7)	167,9 (+/-20,7)	165,8 (+/-2,4)

Figure S2. Effect of the expression of PTC modification enzymes on *Δ10* strain doubling times at 37°C. *E. coli* WT (MG1655), Δ0, and Δ0 strain expressing plasmid-borne native or mutated PTC modification enzymes were grown in 2xYT or LB medium at 37°C. Average doubling times (g) and standard deviations were calculated as above. *WT* and *Δ0* average g values are shown in a dotted line for reference. Panel **A**: comparison of the expression of native RlmE, RluC, RlmN, RlmB, RlmKL, RluB, RluE, RluF, or RlmM in Δ0. Panel **B**: comparison of the expression of native RluC, RlmN, or RlmB with catalytically inactive RluC(D144A), RlmN(C355A), or RlmB(E198A) variants, respectively. Panel **C**: comparison of the expression of native RlmKL hybrid protein with either L- (RlmKL(N309A)), K- (RlmK(D568A)L) or both (RlmK(D568A)L(N309A)) domains mutated version. Also, the native RlmL, mutated RlmL(N309A) as well as RlmK domains were introduced to the Δ0 strain individually.

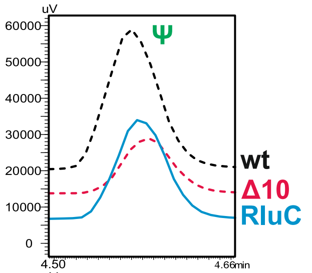

	Ψ(n)	rel. %
wt	9	100
Δ10	4	44
RluC	7	78
RluC (D144A)	4	44

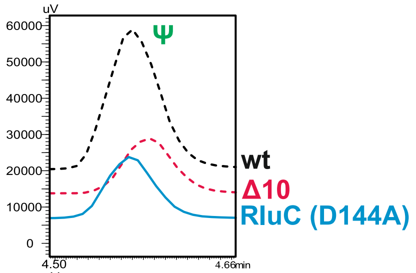

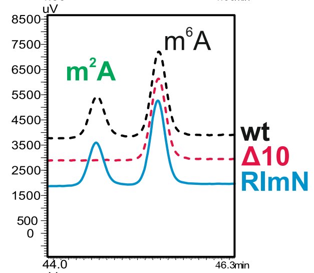

	m^{2}A(n)	rel. %
wt	1	100
Δ10	nd	nd
RlmN	1	100
RlmN (C355A)	nd	nd

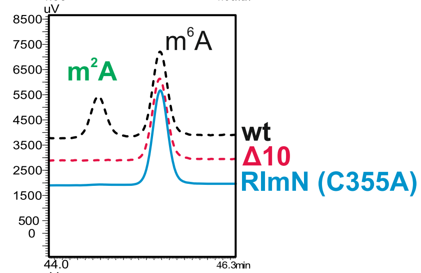

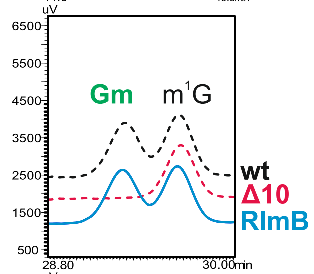

	Gm(n)	rel. %
wt	1	100
Δ10	nd	nd
RlmB	1	100
RlmB (E198A)	nd	nd

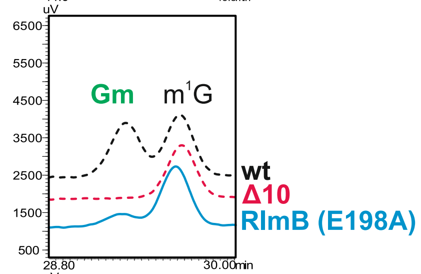

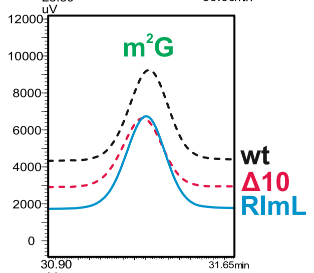

	m^{2}G(n)	rel. %
wt	2	100
Δ10	1	50
RlmL	2	100
RlmL (N309A)	1	50

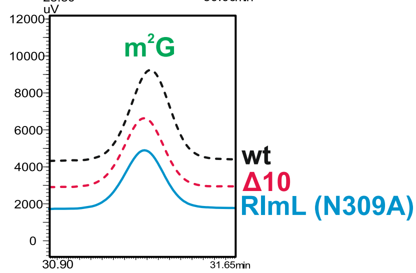

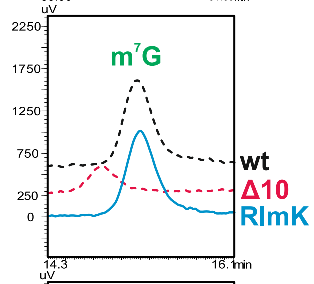

	m^{7}G(n)	rel. %
wt	1	100
Δ10	nd	nd
RlmK	1	100

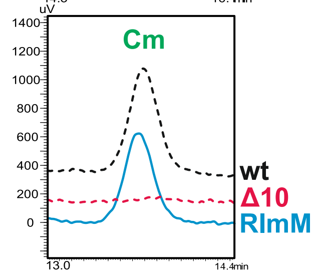

	Cm(n)	rel. %
wt	1	100
Δ10	nd	nd
RlmM	1	100

Figure S3. 50S subunit nucleoside composition of *Δ10* strain expressing native or mutant RlmB, RlmK, RlmL, RlmM, RlmN, or RluC.

50S subunits were purified from mature 70S ribosomes, rRNAs (23S and 5S) were extracted and nucleosides were analyzed as specified above by high-performance liquid chromatography (HPLC). Nucleoside peaks are shown on the left side. Corresponding peak areas of add-back strains relative to the *WT* 23S rRNA control are shown as blue columns. Relative nucleoside content of nonfunctional ME expression strains are as grey columns. Nucleoside corresponding to the respective enzyme is marked by red asterisk.

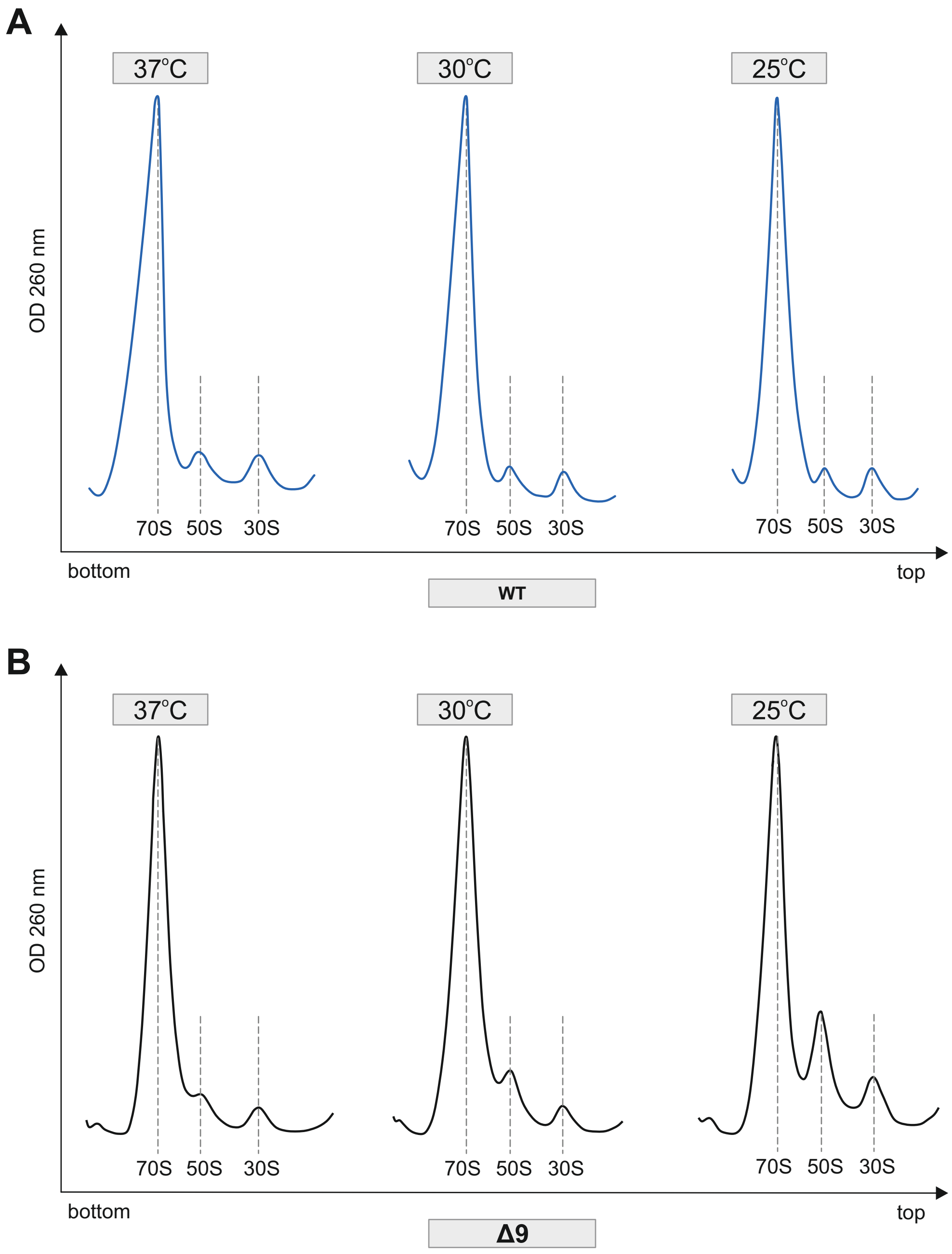

Figure S4. Ribosome profiles of *WT* and *Δ9* strains in the late-log growth phase.

E. coli WT (panel A) and *Δ9* (panel B) were grown into late-log phase (OD_{580} 1.4 - 1.5) at 37°C, 30°C, and 25°C. Cells were lysed and ribosome particles analyzed by sucrose gradient centrifugation.

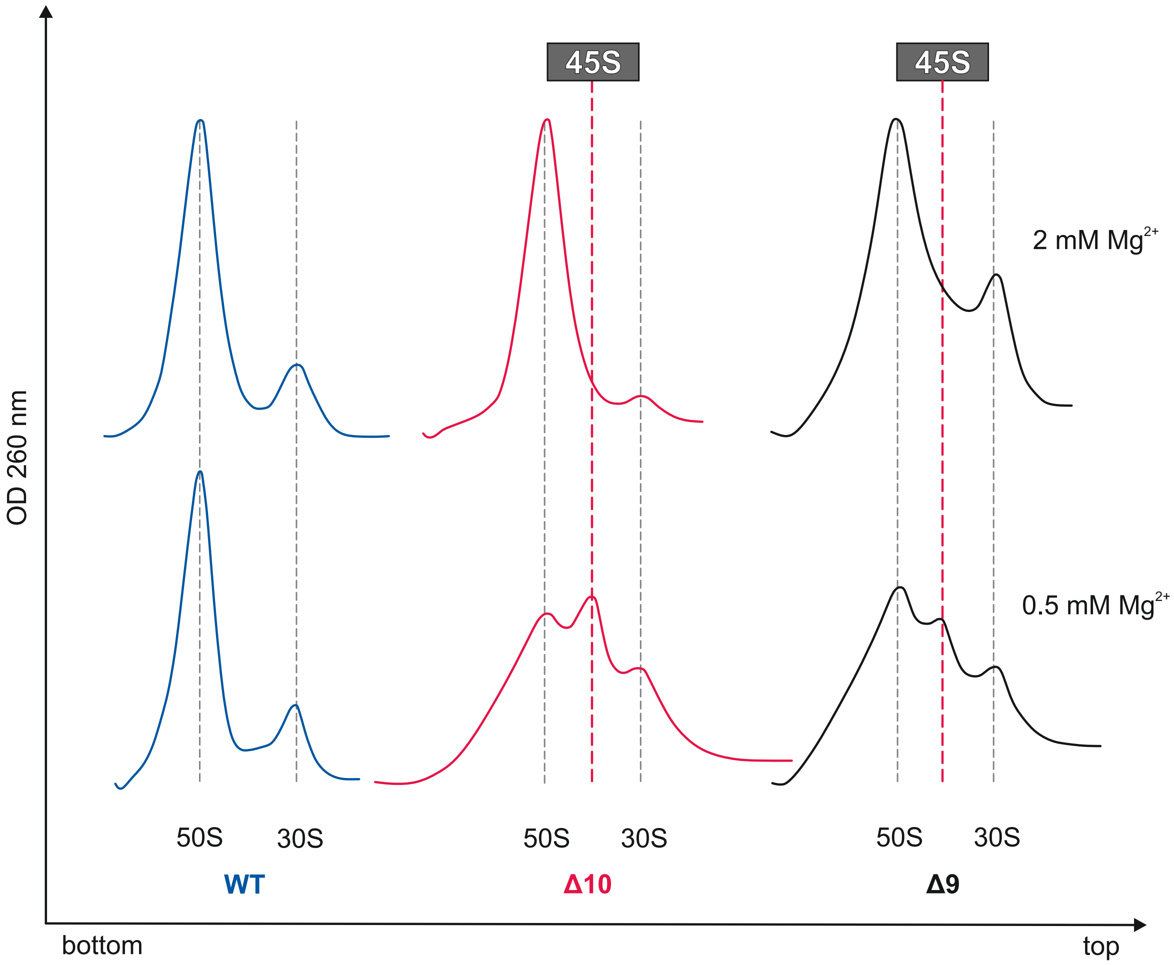

Figure S5. The sedimentation profiles of *in vivo* 50S assembly intermediates from *WT, Δ10,* and *Δ9* cells under different Mg^{2+} concentrations.

Free 50S subunit fractions from sucrose density gradient centrifugation (in the presence of 10 mM Mg^{2+}) were analyzed further by 10% to 25% sucrose density gradient centrifugation in the presence of 2 mM or 0.5 mM Mg^{2+} (Materials and Methods). Ribosome profiles were recorded at OD 260 nm and representative profile from at least two repeats is shown. 45S approximates the sedimentation coefficient for the intermediate-size particles and the 30S subunit is an impurity whose fraction ford not change with [Mg^{2+}].

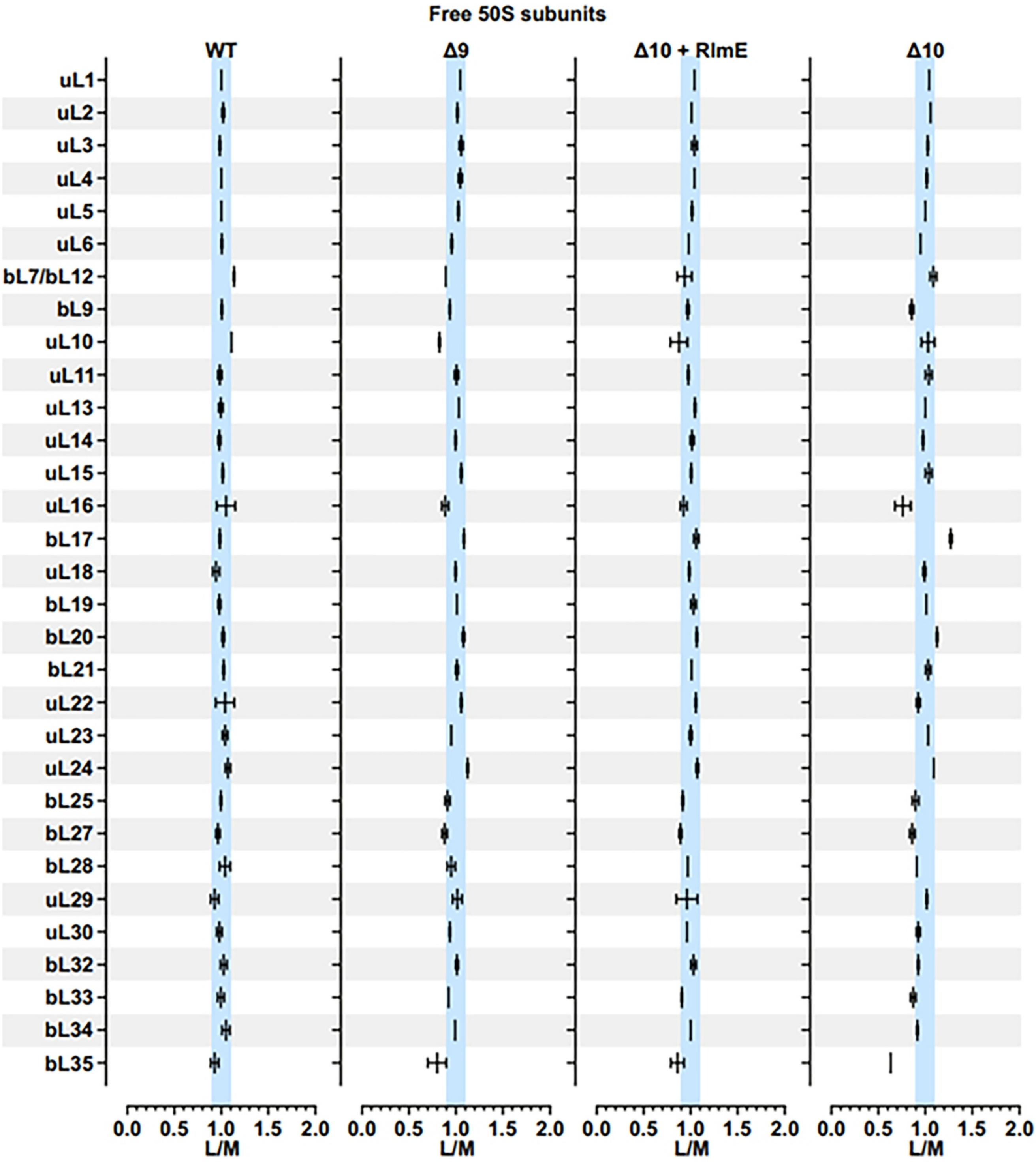

Figure S6. R-protein composition of free 50S subunits.

Free 50S subunits from *MG1655*, *Δ9, Δ10, or Δ10* strains expressing RlmE (*Δ10+RlmE*) were mixed in a 1:1 ratio with "medium" labeled reference 70S ribosomes (from *E. coli MG1655-SILAC* strain grown in MOPS medium supplemented with "medium"-labeled arginine and lysine) for r-protein quantification using LC-MS/MS. Proteins bL31A, bL31B, bL36A, and bL36B were not quantified due to insufficient number of unique peptides. The relative quantities of 31 r-proteins are presented as the L/M ratio (L = sample; M = reference). L/M ratios are normalized against the average L/M ratio of all 50S r-proteins. The blue box marks the ±10% range of the L/M ratio. The values shown in the figure are the means of two independent biological experiments with a standard deviation.

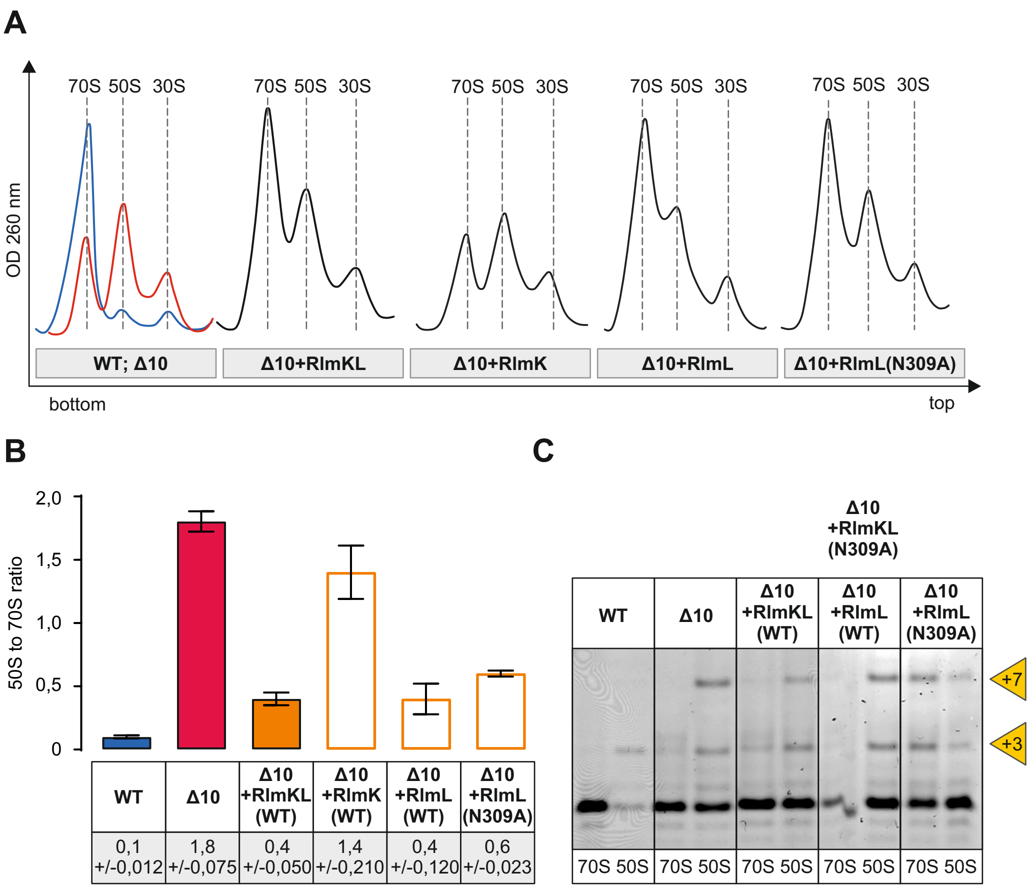

Figure S7. Effect of native RlmKL and individual K and L domains on the ribosome biogenesis phenotype of the Δ10 stain.

E. coli WT (MG1655), Δ10, as well as Δ10 strains expressing plasmid-borne native RlmKL, individual RlmK, RlmL or mutant RlmL (N309A) were grown into late log phase (OD_{600} 1.4 – 1.6) at 37°C and were analyzed as described in the legend of Figure 5. Representative ribosome profiles of at least three independent samples are shown in **panel A**. Peak areas corresponding to 70S ribosomes and free 50S subunits were quantified, and 50S to 70S ratios are shown in **panel B**. 5´ends of rRNA from the 70S and 50S fractions were mapped by primer extension analysis. (**panel C**). +3 and +7 refer to extra nucleotides at the 23S rRNA 5` end.

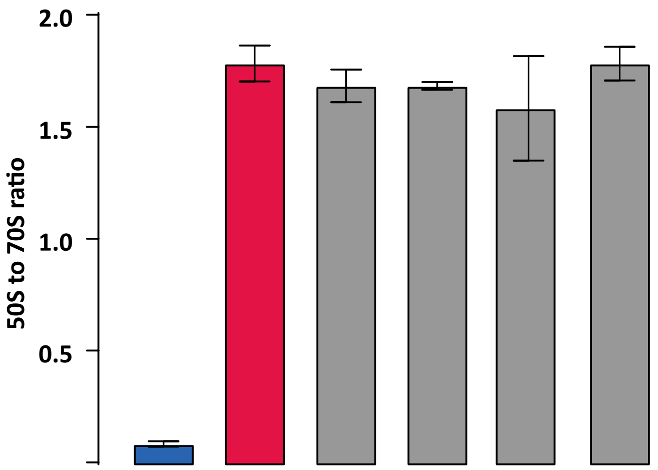

WT	Δ10	Δ10			
		+RlmM (WT)	+RluB (WT)	+RluE (WT)	+RluF (WT)
0,1 (+/-0,012)	1,8 (+/-0,080)	1,7 (+/-0,072)	1,7 (+/-0,016)	1,6 (+/-0,233)	1,8 (+/-0,075)

Figure S8. Effect of modification enzymes on *Δ10* strain ribosome biogenesis phenotype.

E. coli WT (MG1655), Δ10, as well as *Δ10* strain expressing plasmid-borne modification enzymes RlmM, RluB, RluE, and RluF were grown at 37°C. Cells were lysed and ribosome profiles were analyzed by sucrose gradient ultracentrifugation. Peak areas corresponding to 70S ribosomes and free 50S subunits were quantified, and 50S to 70S ratios are shown. At least two biological replicates with two technical replicates were used for calculations with standard deviations shown.
